# Effect of Different Surface Treatments of Lithium Disilicate on the Adhesive Properties of Resin Cements

**DOI:** 10.3390/ma14123302

**Published:** 2021-06-15

**Authors:** Shifra Levartovsky, Hilla Bohbot, Keren Shem-Tov, Tamar Brosh, Raphael Pilo

**Affiliations:** 1 Department of Oral Rehabilitation, The Maurice and Gabriela Goldschleger School of Dental Medicine, Sackler Faculty of Medicine, Tel Aviv University, Tel Aviv 6997801, Israel; Hillab8@gmail.com; 2 Department of Oral Biology, The Maurice and Gabriela Goldschleger School of Dental Medicine, Sackler Faculty of Medicine, Tel Aviv University, Tel Aviv 6997801, Israel; kerenrst77@gmail.com (K.S.-T.); tbrosh@tauex.tau.ac.il (T.B.); 3 Head Department of Oral Biology, The Maurice and Gabriela Goldschleger School of Dental Medicine, Sackler Faculty of Medicine, Tel Aviv University, Tel Aviv 6997801, Israel; rafipilo@gmail.com

**Keywords:** lithium disilicate, shear bond strength, light cured resin cement, hydrofluoric acid

## Abstract

The aim of the current study was to evaluate the influence of hydrofluoric (HF) acid concentration and conditioning time on the shear bond strength (SBS) of dual cure resin cement to pressed lithium disilicate ceramic compared to treatment with an Etch and Prime self-etching glass-ceramic primer (EP). A total of 100 samples of pressed lithium disilicate (IPS e.max Press, Ivoclar Vivadent) were randomly divided into five groups (n = 20) according to surface treatment: two different concentrations of HF (5% or 9%), for different durations (20 or 90 s), or treatment with EP. Adhesion of light-cured resin cement to the treated surface was tested by the SBS test. The substrate surfaces of the specimen after failures were examined by SEM. Data were analyzed using Weibull distribution. The highest cumulative failure probability of 63.2% of the shear bond strength (η parameter) values was in the 9% HF −90 s group (17.71 MPa), while the lowest values were observed in the 5% HF −20 s group (7.94 MPa). SBS values were not affected significantly by the conditioning time (20 s or 90 s). However, compared to treatment with 5% HF, surface treatment with 9% HF showed a significantly higher η (MPa) as well as β (reliability parameter). Moreover, while compared to 9% HF for 20 s, EP treatment did not differ significantly in SBS values. Examination of the failure mode revealed a mixed mode of failure in all the groups. Within the limits of this study, it is possible to assume that IPS e.max Press surface treatment with 9% HF acid for only 20 s will provide a better bonding strength with resin cement than using 5% HF acid.

## 1. Introduction

Restoring teeth with full-coverage metal-ceramic crowns has been the gold standard for more than five decades, although compared to natural teeth; they often present a compromised aesthetic appearance [1]. Therefore, a search for an ideal dental material that transmits and refracts light like a natural tooth has led to the development of all-ceramic restorations. Currently, all-ceramic systems can be categorized into two main groups: those based on oxide ceramics, such as zirconia, which have high mechanical strength, and those based on silica ceramics, such as lithium disilicate, which have reduced mechanical properties but better translucency and aesthetic results, even compared to modern cubic/tetragonal high translucency zirconia [2,3].

Lithium disilicate can be conventionally or adhesively cemented to tooth structure. The choice of cementation, conventional or adhesive, should be in correspondence with the abutment dimension. Conventional cementation is associated with technique simplicity, whereas adhesive cementation is associated with a technique-sensitive multistep procedure that is problematic in cases of subgingival margins [4]. The clinical success of adhesive ceramic restorations is heavily dependent on luting resin cement and cementation procedures, including ceramic surface treatment [5,6,7,8].

Surface treatment of lithium disilicate is attained by combined hydrofluoric (HF) acid and subsequent silane application. During the cementation procedure, chemical bonds and micromechanical interlocking are formed at the resin-ceramic interface. Micromechanical retention is provided by HF acid etching of the ceramic surface, while chemical coupling is provided by the application of a silane coupling agent [9,10,11]. The HF removes the glass matrix and the second crystal phase (lithium orthophosphate), creating a rough surface with irregularities within the lithium disilicate for bonding [12,13]. Furthermore, the silane coupling agent, promotes the chemical adhesion between the silica in the glass phase of the ceramics and the organic phase (the methacrylate groups) of the resin cement through siloxane bonds [14,15]. A variety of silane primers have been marketed, but the majority contain dilute (2–5 wt%) alcoholic solutions of 3-methacryloxypropyltrimethoxysilane (MPTMS), either as two-bottle systems for hydrolytic activation prior to application or as a single-bottle prehydrolyzed form.

According to the manufacturer’s directions of use, etching the lithium disilicate surface is recommended with 5% HF acid for 20 s [16]. However, in many studies, a higher concentration (9.5–10%) of HF acid has been used [13,17,18,19]. This higher concentration corresponds to the recommended surface treatment of feldspathic ceramic, which is usually 9–10% HF [20,21]. Studies examining the effect of overetching (higher acid concentration and/or longer exposure time) on the bond strength (SBS) of lithium disilicate to resin cement have led to contradictory results. Prochnow et al. [22] evaluated the effects of different HF acid concentrations on the cyclic load-to-failure of CAD-CAM lithium disilicate crowns cemented by resin cement to dentin and found no negative effect of the different HF concentrations. Fonzar et el. [18] studied the influence of HF acid concentration and etching time on the micro-shear bond strength (µSBS) of lithium-silicate glass ceramics to resin cements and demonstrated that while material and HF concentration influenced the µSBS, the etching time was not an influential factor. Their conclusion was that the most effective etching treatment for lithium disilicate was the use of 4.9% HF for 20 s. In contrast, Sudré et al. [19] evaluated the effect of different HF acid concentrations on the surface roughness of IPS e.max Press and on the bond strength to resin cements and showed that HF concentration and exposure time significantly affect the SBS between IPS e.max Press and resin cement. Their conclusion was that the highest roughness value was obtained with 10% HF for 20–40 s, but that the highest bond strength value was produced with 5% HF for 40 s. When comparing IPS e.max CAD, IPS Empress CAD and IPS e.max Press, Veríssimo et al. [23] demonstrated that for IPS e.max Press, 10% HF for 60 s showed significantly higher bond strength to resin cement, whereas for IPS e.max CAD and IPS Empress CAD, the recommendation was 5% HF for 20 s.

Several years ago, an alternative product termed Monobond Etch and Prime (Ivoclar Vivadent, Schaan, Liechtenstein), which is a self-etching ceramic primer (EP) containing the etching agent and silane components in the same vial, was launched for simultaneous etching and silanization. The product is free of HF acid, which has been replaced by a water/alcohol solution of tetrabutyl ammonium dihydrogen trifluoride (TADF) etchant. The performance of this self-etching primer was compared to the conventional surface treatment method combining prior HF acid application and subsequent silanization and reported to be similar [24,25,26].

Currently, there are inconclusive data among studies on the preferable method to treat lithium disilicate prior to cementation by resin cement, concerning a surface treatment with 5% compared to 9% HF, as well as the amount of conditioning time that is needed. Thus, the aim of the current study was to evaluate the influence of HF acid concentration (5% vs. 9%) and conditioning time (20 vs. 90 s) on the shear bond strength of dual cure resin cement to pressed lithium disilicate ceramic compared to treatment with a self-etching glass-ceramic primer (EP). Our null hypothesis was that different concentrations of HF acid or conditioning times as well as treatment with a self-etching glass-ceramic primer (EP) will not affect the bond strength values.

## 2. Experimental Section

A total of one hundred pressed lithium disilicate (IPS e.max Press, Ivoclar Vivadent, Schaan, Liechtenstein) ingots, 12 mm in diameter and 10 mm in thickness (A2 HT) one surface per ingot, comprised the study sample. The samples were randomly divided into 5 groups, 20 ingots each, according to the surface treatment, as shown in Table 1; 5% HF acid (IPS Ceramic Etching Gel, Ivoclar Vivadent, Schaan, Liechtenstein) or 9% HF acid (Bisco, Schaumburg, IL, USA) was applied with a microbrush (Dentsply, New York, NY, USA) for 20 or 90 s, washed with water spray for 30 s, and then air-dried for 30 s. Afterwards, a thin layer of silane-based primer (Monobond Plus, Ivoclar Vivadent, Schaan, Liechtenstein) was applied with a microbrush (Dentsply, New York, NY, USA) and left to react for 60 s. The remaining excess was dispersed with a strong stream of air for 10 s. In group 5 (Table 1), EP (Monobond Etch and Prime, Ivoclar Vivadent, Schaan, Liechtenstein) was applied for 60 s according to the manufacturer’s recommendations. Every ingot was embedded in a designated metal holder after surface treatment (Table 1) with the outer treated surface being exposed.

Polyethylene molds (5 mm diameter, 10 mm length) were used to fabricate standardized resin cylinders. Two-thirds of the mold was initially filled with light cure resin cement (IPS Empress Direct, A1, Ivoclar Vivadent, Schaan, Liechtenstein) and light cured for 40 s with an LED-curing unit (1200 mW cm^−2^, Bluephase, Ivoclar Vivadent, Schaan, Liechtenstein). The other one-third of the mold will be subsequently filled with dual cure luting composite resin cement (Variolink Esthetic DC, Ivoclar Vivadent, Schaan, Liechtenstein) prior to adhesion to the lithium disilicate ingots. Variolink DC was preferred over Variolink LC because the latter is restricted to restorations of low material thickness and sufficient translucency, whereas the former cover all indications.

A special jig was built for the cementation process to verify perpendicularity. Every ingot was embedded in a designated metal holder after surface treatment (Table 1) with the outer treated surface being exposed. The polyethylene mold filled with the resin cement was placed in a metal holder opposite to the ingot and then manually transported towards the center of the ingot until it stopped. Then, the surplus of the resin cement was removed, and the resin cement in the mold, which was closely adhered to the treated surface of the ingot, was light cured for 40 s with an LED curing light (Bluephase, Ivoclar Vivadent, Schaan, Liechtenstein).

### 2.1. SBS Test

**Figure 1:**
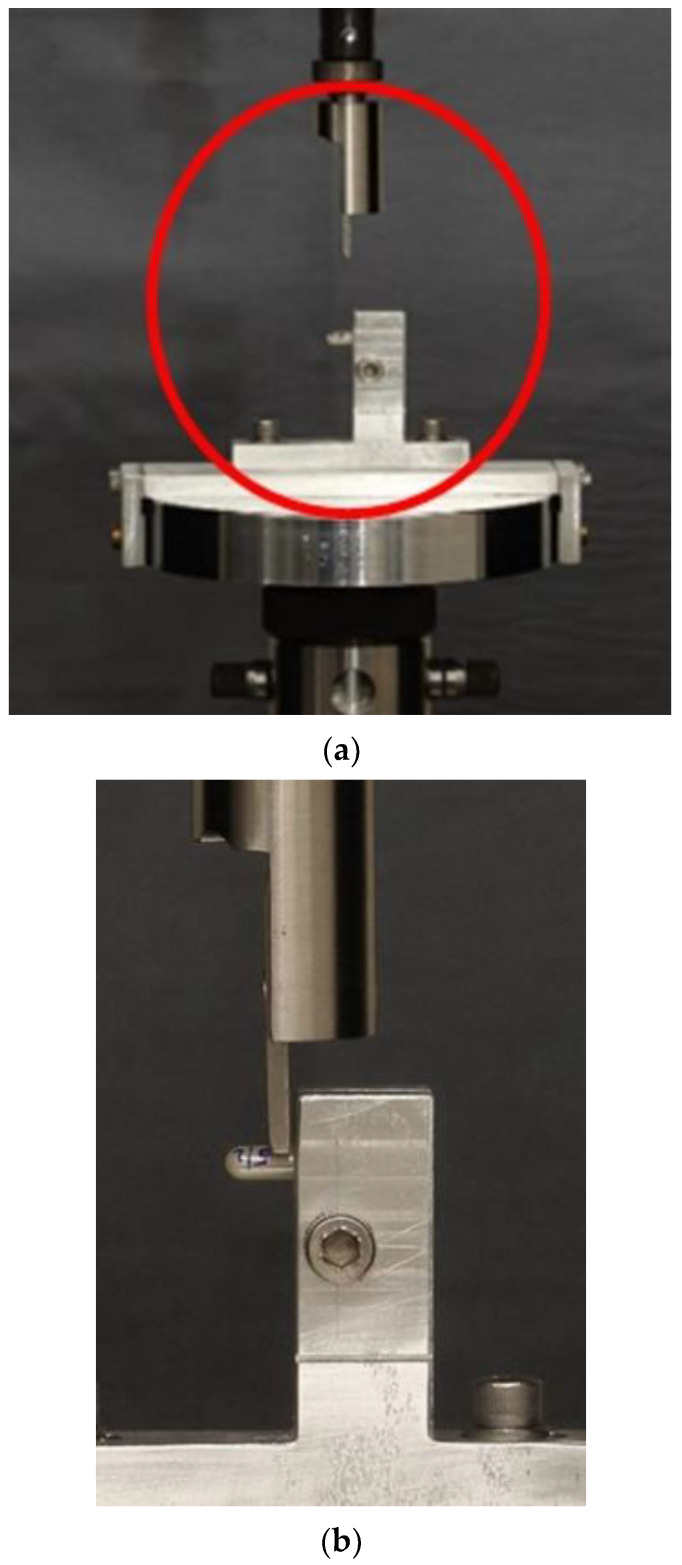
Sample mounted in the universal testing machine (**a**), shear force application at the ingot-cement interface (**b**).

Post cementation, all ingot-resin cement specimens were stored at room temperature for 24 h and then mounted in a universal testing machine (Instron, Model 4502, Instron Corp., Buckinghamshire, UK) for shear bond strength (SBS) testing via a knife-edge rod, according to ISO 29022:2013, at a crosshead speed of 0.5 mm/min of continuous loading until fracture or debonding occurred, and the load was recorded (Figure 1a,b). The shear force value (MPa) was calculated by dividing the force at debonding by the total surface area of each sample.

### 2.2. Failure Mode

Examination of the debonded surfaces of the samples was performed microscopically at ×10 and ×16 magnifications (Kaps ENT SOM Microscope, Asslar, Germany). Failure was classified as adhesive (between the resin cement and the lithium disilicate), cohesive (within the resin cement) or mixed mode (part between the cement and the ceramic and part within the cement).

### 2.3. SEM

Two samples from each group were randomly selected and examined using scanning electron microscopy (SEM, Joel, Tokyo, Japan) (Quantum 2000, high vacuum mode, following gold sputter coating). The acquisition conditions were as follows: 25 kv, 90 µA, at ×2000 magnification.

### 2.4. Statistical Analysis

The data for the shear bond strength (SBS) values were analyzed using a Weibull distribution, which gave the probabilities of failure occurrence; variables: η (characteristic life)—is the highest cumulative 63.2% failure probability of the shear bond strength (SBS) values. Β—the slope of the curve plot. The significant difference was calculated within a confidence interval of 95%.

## 3. Results

### 3.1. SBS test

**Figure 2:**
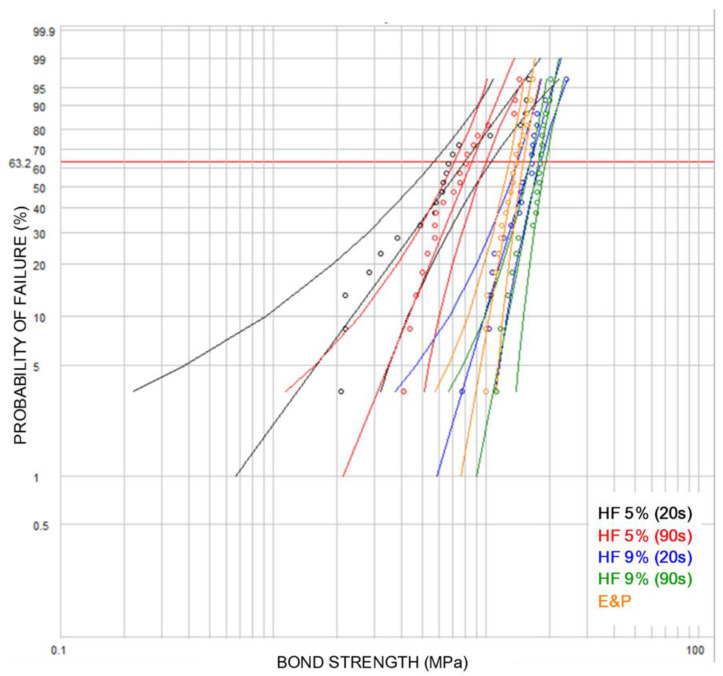
Weibull graphs for the treatments with 5% HF (20 and 90 s), 9% HF (20 and 90 s) and Etch and Prime (EP). Reliability is indicated by the slope of the graph. The characteristic life is indicated by the intersection of the graph with 63.2% probability (horizontal line).

The results of the shear bond strength test according to the Weibull distribution (Weibull parameters) are presented in Table 2 and Figure 2.

The treatment with the highest characteristic life (η parameter in MPa) was the 9% HF −90 s group (17.71 MPa), while the lowest values were observed in the 5% HF −20 s group (7.94 MPa). No statistically significant differences in the characteristic life were found using a 20 or 90 s conditioning time in either the 9% HF or 5% HF groups. SBS values thus were not affected significantly by the conditioning time.

However, compared to treatment with 5% HF, surface treatment with 9% HF showed significantly higher SBS, as indicated by the η parameter, while, EP treatment did not differ significantly in comparison to 9% HF for 20 s (Table 2).

Similarly, the reliability (β parameter) of 9% HF was significantly higher than that of treatment with 5% HF.

### 3.2. Failure Mode

Examination of the failure mode after debonding of the samples revealed mixed mode failure in all the samples, meaning that part of the ceramic surface was always covered with the cement and part of the surface was denoted from the cement and revealed the lithium disilicate surface.

### 3.3. SEM

**Figure 3:**
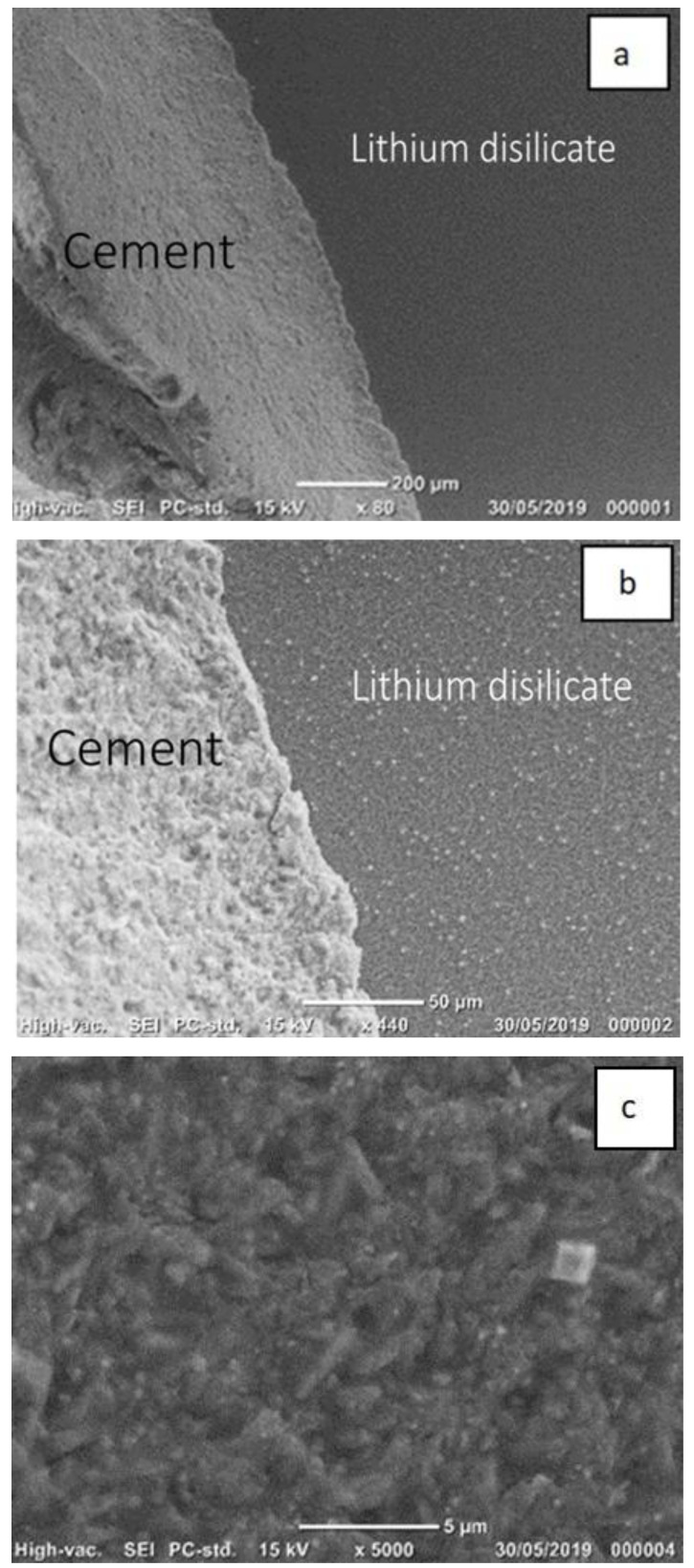
(**a**–**c**)—Scanning electron images of the mixed mode failure in a representative sample of Group 2 (5% HF for 90 s). (**a**,**b**) The lithium disilicate surface (black area) is covered partly with cement (white area) (×440). (**c**) Lithium disilicate crystals are visible following the removal of the glassy phase (×5000).

**Figure 4:**
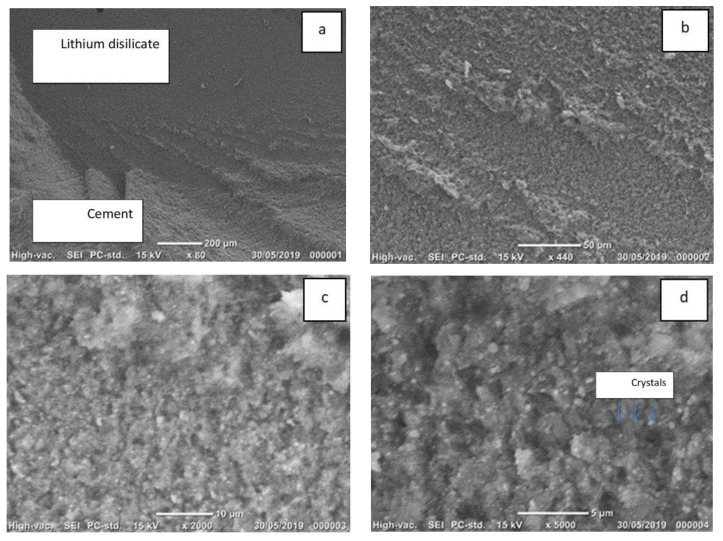
(**a**–**d**) Scanning electron images of the mixed mode failure in a representative sample of Group 4 (9% HF for 90 s). (**a**,**b**). Lithium disilicate surface covered partly with cement (×440) (**c**,**d**). The higher acid concentration caused further degradation and more surface irregularities in the ceramic. Lithium disilicate crystals are visible at lower (×2000) and higher (×5000) magnifications following the removal of the glassy phase.

**Figure 5:**
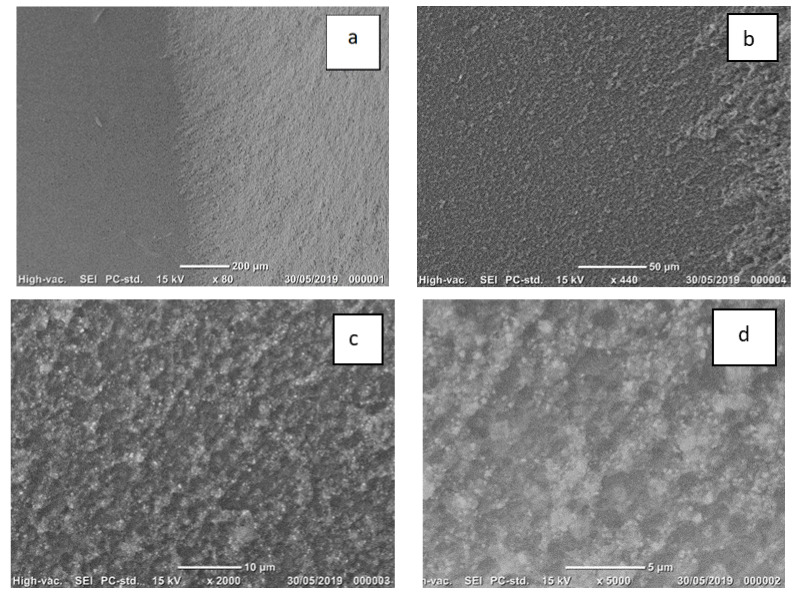
(**a**–**d**)—Scanning electron images of the mixed mode failure in a representative sample of Group 5 (EP). (**a**,**b**). In the uncovered ceramic surface, EP seems to produce fewer pronounced irregularities than HF (×440). (**c**,**d**). Lithium disilicate crystals are scarcely evident (×5000).

Examples of SEM photomicrographs of the failure modes are given in Figure 3, Figure 4 and Figure 5. The only pattern was mixed mode with the lithium disilicate surface, partly exposed and partly covered with cement. The white area in the micrographs presents the resin cement layer, while the dark area presents the lithium disilicate surface area. The SEM micrographs showed that the lithium disilicate exposed surface area in the EP group (Figure 5) had fewer irregularities than those in the 5% HF (Figure 3) and 9% HF (Figure 4) groups. The higher acid concentration (Figure 4) caused further degradation and more surface irregularities in the ceramic. In the denoted cement areas of 5% HF (Figure 3) and 9% HF (Figure 4), lithium disilicate crystals were visible following the removal of the glassy phase at high magnifications.

## 4. Discussion

The results of the present study have led to partial rejection of our null hypothesis; HF acid concentration did affect the bond strength values, while surface treatment with 9% HF showed significantly higher SBS than 5% HF treatment. This is reflected by both significantly higher η (MPa) values and better reliability (β parameter). However, the SBS values were not affected significantly by the conditioning time in either the 5 or 9% HF groups. Even if IPS e.max CAD and Press have initially similar material composition, the handling and process steps differ and by means the end result material properties differ, that includes as well other lithium silicate based glass ceramics. This is the reason that the results must be related to IPS e.max Press only.

Lithium disilicate microstructure consists of small, interlocking, densely packed, needle-like lithium disilicate crystals that are randomly oriented, with the addition of much smaller secondary lithium orthophosphate crystals. HF etching produces a porous surface by dissolving and removing the glassy matrix containing silica and silicates. The secondary crystal phase provides greater micromechanical retention of the surface [27]. 9% HF might have further effect on that second phase, thus providing greater micromechanical retention as compared to 5% HF.

Some of these findings are consistent with those previously reported. Fonzar et al. tested the influence of HF acid concentration and etching time on the micro-shear bond strength (µSBS) of lithium-silicate glass ceramics (zirconia-reinforced lithium-silicate) and IPS e.max CAD to resin cements [18]. Similar to our results, it was demonstrated that adhesion was affected by the HF acid concentration but not by the etching time. Unlike our findings, their conclusion was that the most effective etching treatment was 4.9% HF for 20 s, especially for zirconia-reinforced lithium-silicate glass ceramics. However, it should be noted that the resin cement used in their study was RelyX Unicem 2, which is self-adhesive resin cement, whereas we used dual cure composite resin luting cement (Variolink Esthetic DC).

Veríssimo et al. [23] compared the effect of HF acid concentration (5% vs. 10%) and conditioning time (20 s vs. 60 s) on the shear bond strength of IPS e.max CAD, IPS Empress CAD and IPS e.max Press to a resin cement. The interaction “acid concentration X ceramic” had a significant effect on the SBS; however, the “ceramic” and “conditioning time” factors did not influence the results. According to their findings, for the lithium disilicate and leucite reinforced CAD/CAM ceramics, 5% HF etching for 20 s was recommended, but for the pressed lithium disilicate ceramic, 10% HF for 60 s showed significantly higher bond strength. This is in accordance with our results, as IPS e.max Press presented the best results with a surface treatment with 9% compared to 5% HF.

The current results, which show that conditioning time did not affect the bond strength values, are not in agreement with other previous reports. Sudré et al. [19] tested the influence of HF acid concentration (5% vs. 10%) and conditioning time (20 s, 40 s, and 60 s) on the surface roughness and micro-shear bond strength of self-adhesive resin cement to IPS e.max Press samples. Although the surface roughness value was influenced by both the acid concentration and exposure time, the bond strength was affected only by the exposure time; in both the 5% and 10% HF groups, the highest bond strength value was obtained with a 40 s conditioning time. However, when the exposure time was increased to 60 s, the surface roughness and the bond strength were reduced, probably due to the partial destruction of the lithium disilicate crystals. In our study, the tested conditioning time was 20 s (as per the manufacturer recommendation) vs. 90 s (as recommended for the surface treatment of feldspathic ceramic), with no significant difference in the SBS. We did not have 40 or 60 s groups to compare.

When recommending a combination of acid concentration and exposure time, other factors that were not tested in the current study should also be evaluated, mainly the strength of the ceramic after acid etching and bonding and its optical properties. Prochnow et al. [22] evaluated the effects of different HF acid concentrations of 3%, 5% or 10% on the cyclic load-to-failure of CAD–CAM lithium disilicate crowns cemented by resin cement and found no negative effect of the different HF concentrations. Similarly, in a previous study by Prochnow et al. [28], the surface roughness and flexural strength of e.max CAD blocks were tested after using distinct HF acid concentrations of 3%, 5% or 10%. No effect of the HF acid concentration on the roughness and mean flexural strength values was found. In both aforementioned studies, machined (CAD) lithium disilicate glass-ceramics were used. Their SEM images indicated that different concentrations of HF acid etching did not change the surface pattern of the hard machined lithium disilicate, and no pullout of crystals could be observed. Their conclusion was that CAD/CAM machining potentially changes the lithium disilicate ceramic surface, and when HF acid etching is applied, no pullout of lithium disilicate crystals occurs. Thus, for machined lithium disilicate glass-ceramics (CAD), the influence of HF acid etching overlaps with the machining process [22]. Moreover, it should be noted that in the Prochnow et al. [22] study, a cyclic load was applied in the center of the occlusal surface of each crown, while in our study, a shear force was applied in the interface between the lithium disilicate ceramic surface and the cement. HF acid concentrations might thus affect the shear bond strength of the ceramic to resin cement, but this not necessarily would be detected in other configurations of force application. Zogheib et al. reported that a longer application time (20, 60, 90 and 180 s) of 4.9% HF negatively affected the 3-point bending strength of partially crystallized lithium disilicate-based glass blocks (IPS e.max CAD) when compared to only the untreated control specimens but not between the HF treatments themselves [29]. The drawback of this study is that the material was tested without adhesive bonding of resin cement, which occludes the irregularities created by the acid and thus diminishes the phenomena of crack propagation from the tension side.

Apparently, different processing techniques (pressing vs. CAD/CAM) of the lithium disilicate ceramic material may influence their surface characteristics and fatigue performance. Schestatsky et al. demonstrated that pressed lithium disilicate monolithic crowns showed better fatigue performance than CAD/CAM milled crowns when adhesively cemented to a dentin-analog material [30].

Based on the current results and the aforementioned studies, our recommended protocol is 5% HF for 20 s, since prolonged conditioning time (90 s) had no effect on the SBS values. Although the 90 s application yielded a higher β parameter and consequently better reliability, the differences were not statistically significant. Regarding the comparison of the SBS values in the current study, between surface treatments by EP versus HF acid + silane, significantly higher SBS was obtained compared to 5% HF and no significant difference compared to 9% HF for 20 s. These results are in agreement with previous studies, meaning that EP provides comparable bond strengths to lithium disilicate to those obtained by the traditional bonding protocol using HF acid etching followed by silane [24,25,30,31,32]. However, some of these studies tested SBS without any aging of the samples [24], as in the current work, and some used thermocycling as aging conditions [25,30,31,32]. Tribst et al. found an even higher bonding strength between ceramic and resin cement when EP was used compared to HF + silane, but this SBS was reduced significantly after thermocycling [26]. Schestatsky et al. showed that surface treatment with EP led to similar fatigue performance when compared to HF + silane application for both CAD and Press processing techniques, but it tended to provide higher mechanical reliability [30]. On the other hand, Dimitriadi et al. demonstrated a significantly lower SBS with EP in all treatments/storage conditions in comparison to HF + silane [33]. When HF was used without silane, similar SBS values were recorded after storage in distilled water at 37 °C for 1 week, but after thermocycling (5000 × 5 °C–55 °C, 20 s dwell time) or accelerated aging conditions (immersion in 100 °C water for 24 h), EP showed even significantly lower SBS than HF without silane. It was demonstrated that the bonding mechanism of EP is related to the interaction of phosphoric monomers and ceramic ions rather than the methacrylate silane bonding to the glass ceramic. After aging, the self-etching silane primer was stable, retaining the original silanol activity; however, the stability of the phosphate comonomer was affected, leading to values even lower than those of the negative control (HF-etched substrate without silane) [33]. In the current study, we tested SBS immediately after storing the samples at room temperature for 24 h without thermocycling. It might be that the aging conditions of the samples would give different results.

The limitation of this study was that, similar to other in vitro studies, a laboratory study design cannot fully reproduce the clinical conditions. Moreover, the samples of the lithium disilicate used were discs and not full crowns. Additionally, only two conditioning times were tested, and the samples were not artificially aged. The current study intentionally aimed to test the specimens without aging to determine the possibility that the differences are attributed to the aging process; however, a combined regime of with and without aging would overcome that issue. Due to these limitations, further in vitro and in vivo research is required.

## 5. Conclusions

Within the limitations of the current study, we can conclude that the highest shear bond strength (SBS) values between IPS e.max Press and dual cure resin cement were obtained with surface treatment with 9% HF. This gives significantly higher and reliable results. SBS values were not affected significantly by the conditioning time (20 s or 90 s). Therefore, it is possible to assume that IPS e.max Press surface treatment with 9% HF for only 20 s will provide a better bonding strength than using 5% HF acid for 20 s, as per the manufacturer recommendation. In addition, SBS values with EP treatment were comparable to those with 9% HF for 20 s.

## Figures and Tables

**Table 1:** The various surface treatments applied to the IPS e.max Press ingots.

Group	Acid Treatment	Conditioning Time (s)	Silane Application
1	5% HF	20	+
2	5% HF	90	+
3	9% HF	20	+
4	9% HF	90	+
5	EP	60	−

HF—hydrofluoric acid; EP—self-etching glass-ceramic primer.

**Table 2:** Results of the shear bond strength test (Weibull parameters).

Treatment	β Parameter	η (MPa) Parameter	95% CI-η	r2-η
5% HF −20s	1.86 ^a^	7.9	10.59–5.78 ^a^	0.9174
5% HF −90s	3.31 ^a^	8.53	10.02–7.10 ^a^	0.8874
5% HF −20s	4.59 ^b^	16.07	18.00–14.05 ^b,c^	0.9566
5% HF −90s	6.84 ^b,c^	17.71	19.15–16.12 ^b^	0.9314
EP	7.56 ^c^	13.95	14.94–12.93 ^c^	0.9493

EP—self-etching glass-ceramic primer; The same letters indicate η values with no statistically significant differences (*p* > 0.05).

## Data Availability

The data presented in this study are available upon request from the corresponding author.
